# Generalized permanent dentition fluorosis severity becomes less evident over time among a birth cohort

**DOI:** 10.3389/froh.2023.1198167

**Published:** 2023-06-30

**Authors:** Steven M. Levy, John J. Warren, Justine L. Kolker, Karin Weber-Gasparoni

**Affiliations:** ^1^ Department of Preventive & Community Dentistry, College of Dentistry, The University of Iowa, Iowa City, IA, United States; ^2^ Department of Epidemiology, College of Public Health, The University of Iowa, Iowa City, IA, United States; ^3^ Department of Operative Dentistry, College of Dentistry, The University of Iowa, Iowa City, IA, United States; ^4^ Department of Pediatric Dentistry, College of Dentistry, The University of Iowa, Iowa City, IA, United States

**Keywords:** epidemiology, fluoride(s), pediatric dentistry, dental fluorosis (DF), changes, longitudinal

## Abstract

**Objectives:**

There are relatively few cohort studies which have examined changes in fluorosis appearance over time, and none of these have assessed changes in generalized fluorosis. In this analysis, we quantified and assessed changes in multiple measures of generalized fluorosis severity through childhood, adolescence, and young adulthood.

**Methods:**

Participants were from the Iowa Fluoride Study, a birth cohort recruited from 1992 to 1995. Permanent dentition fluorosis exams were carried out at ages 9, 13, 17, and 23 years using the Fluorosis Risk Index (FRI). Generalized fluorosis was assessed using mean FRI scores at the tooth- and person-level as well as a five-category measure of generalized fluorosis. Generalized fluorosis prevalence and severity was summarized at each time point and differences in adjacent time points were assessed using gamma statistics, signed-rank tests, and plotting changes in generalized fluorosis between adjacent time points.

**Results:**

We observed a statistically significant decline in the percentage of non-zero mean FRI scores at later exam ages at both the person- and tooth-levels. Based on our five-category generalized fluorosis measure, there were 34.0%–54.1% of participants with generalized fluorosis at baseline for each tooth group, and these percentages declined to 8.9%–27.2% at the age 23-year exam.

**Conclusions:**

We observed a statistically significant decline in generalized fluorosis severity scores and overall prevalence at later exam ages across all three measures of generalized fluorosis severity. This trend should be accounted for when estimating the prevalence of fluorosis in a population using fluorosis severity data collected in children and adolescents.

## Introduction

Fluoride, most often consumed through community water fluoridation and toothpaste, has been shown to be a very effective means of caries prevention (1). The only proven downside to fluoride for caries prevention is dental fluorosis (1). This occurs during amelogenesis early in life when excess fluoride interferes with the normal early secretion of matrix proteins (2). Consequently, the enamel is hypo-mineralized and can result in white discoloring of the teeth that is mild and only of cosmetic concern in most cases (3–5).

Recent analyses of dental examination data from the National Health and Nutrition Examination Survey (NHANES) have shown higher prevalence and severity of fluorosis in U.S. children and adolescents than reported previously. However, there was some evidence of limitations with the measurement (6–8). The authors of the data quality report which accompanied the 2011–2016 NHANES data suggested that these results were implausible under the assumption that fluorosis severity remained the same after tooth eruption (7). One small cohort study from the 1970s demonstrated a decline in dental fluorosis severity over time (9). Additionally, more recent cohort studies that examined fluorosis in children, adolescents, and young adults showed a decline in fluorosis prevalence and/or severity over time. These recent reports included participants from Hong Kong (10), South Australia (11), and the Iowa Fluoride Study (IFS) (12).

In previous papers, the emphasis was strictly on the buccal surfaces with the most severe fluorosis, which did not capture the full range of fluorosis cases. Thus, in order to assess changes across the full range of fluorosis severity throughout childhood, adolescence, and young adulthood, IFS data were used to quantify changes in: (1) the mean Fluorosis Risk Index (FRI) score and (2) the presence of mild or moderate fluorosis on at least half of the teeth in each of three tooth groups–early-erupting, maxillary incisors, and late-erupting teeth. The rationale for the latter grouping was that maxillary incisors are the most esthetically important teeth, such that changes in fluorosis appearance would be most meaningful for these teeth and changes in early-erupting and late-erupting teeth were explored to assess whether time since eruption impacted the changes in fluorosis appearance.

## Materials and methods

The IFS recruited mothers and newborns from eight maternity wards across the state of Iowa from 1992 to 1995. The primary goal of the study was to collect data on children's fluoride intake and quantify the associations between fluoride intake and dental (and later bone) health. The IFS was approved by the Institutional Review Board of the University of Iowa and parental consent and child assent (and later child/young adult consent) were obtained at each stage of the study. Although the data collection techniques and much of the statistical analysis methods used here have been described in detail in a recent publication from this study (12), a summary is provided here.

Examinations for dental caries and fluorosis were carried out on the permanent dentition at ages 9, 13, 17, and 23 years by trained and calibrated examiners (12). In order to differentiate between fluorosis and other enamel defects, examiners used Russell's criteria in addition to texture, location, and color of each lesion (13).

At the tooth level, examiners scored three horizontal buccal zones and the incisal edge/occlusal table of each tooth using the Fluorosis Risk Index (14). The FRI was chosen as the primary index in the overall IFS study to allow researchers to relate detailed fluoride intake data at specific time periods early in life to the presence of fluorosis on the different FRI tooth zones in the permanent teeth, including early- and late-erupting teeth and early- and late-developing tooth zones. Briefly, the FRI categories for each small zone are: no fluorosis (score of 0), questionable fluorosis—less than half of the zone was affected by white striations (1), positive fluorosis—greater than half of the zone was affected by white striations (2), severe fluorosis—pitting, staining, or deformity (3), and non-fluoride opacity without fluorosis (0). (Note that the FRI's “questionable” category includes many zones with fluorosis affecting less than 50% of the zone, as well as some zones that do not have fluorosis.) Kappa scores at each wave based on the subset with duplicate exams were used to assess inter-rater reliability among the three examiners at each age, but intra-examiner reliability was not assessed (15).

Since examinations were conducted during the ongoing eruption of the permanent dentition, we reported on teeth which were erupted and scored for most participants (12). At age 9, this included early-erupting teeth (permanent incisors and first molars) and maxillary incisors (the most visible teeth, a subset of the early-erupting teeth). At ages 13, 17, and 23, we included the early-erupting teeth, maxillary incisors, and late-erupting teeth (canines, premolars, and second molars).

In these analyses, fluorosis severity was assessed using comparisons of the mean FRI score at the person- or tooth-levels between dental exams which were adjacent in time. At the person-level, the second highest FRI score, as used in our previous study, does not differentiate between an individual who has only one zone on two different teeth with definitive fluorosis and another individual with widespread definitive fluorosis, or generalized fluorosis. Therefore, to study the change in fluorosis severity in a more generalized way, we used the intra-participant mean FRI score over all zones in the three tooth groups: early-erupting teeth, maxillary incisors, and late-erupting teeth.

At the tooth level, we calculated mean FRI scores of the available zones on each tooth (maximum of four zones). We separately examined trends for changes in the early- and late-erupting teeth, as well as the maxillary incisors, from one exam to the next.

In order to summarize mean fluorosis severity over the four time points, mean FRI scores were grouped into four categories with the goal of obtaining clinically-meaningful categories which each contained a reasonable number of participants, especially at the age 9 exam (baseline). The outcome variables at the person- and tooth-levels have been categorized into the following mean FRI values or ranges: 0, 0.01–0.50, 0.51–1.00, and 1.01–3.00.

One limitation of the mean FRI score was that participants with a few zones with FRI scores of 2 or 3 could have a similar mean FRI score to a participant who has many zones with an FRI score of 1. To address this concern, and assess changes across different fluorosis presentations, we calculated an additional person-level definition of generalized fluorosis based on common clinical presentations, which provides a more direct definition of generalized fluorosis. We then examined changes in these levels of generalized fluorosis over time. This additional person-level generalized fluorosis measure grouped participants into the categories described in Table 1.

**Table 1:** Summary of generalized fluorosis categories, listed in order from most severe generalized fluorosis to no fluorosis.

Category	Description
Generalized Positive Fluorosis	At least 50% of available teeth in the tooth group have a maximum FRI score of at least 2.
Generalized Fluorosis (not meeting positive threshold)	Participant does not qualify for the generalized positive fluorosis category, and at least 50% of available teeth in the tooth group have a maximum FRI score of at least 1
Any Positive Fluorosis	Participant does not qualify for either of the generalized fluorosis categories above, but has some positive fluorosis (FRI score of at least 2)
Any Questionable Fluorosis	Participant does not qualify for any of the categories above, but has some questionable fluorosis (FRI of 1)
No Fluorosis	No fluorosis—FRI scores all 0

When calculating these outcome variables and analyzing results across all four time points, we limited the calculation of person- and tooth-level mean fluorosis severity to zones that were examined at all ages. However, when analyzing pairwise differences, we limited the calculation of these fluorosis severity measures to zones examined at both ages.

After summarizing the number of participants and teeth in each mean FRI category and generalized fluorosis category at each age, we used Goodman and Kruskall's gamma statistics (16) and the standard asymptotic standard error at the person level to quantify the trend in fluorosis severity for each tooth group and test for a statistically significant change in mean FRI score category or generalized fluorosis category with age. At the tooth level, we used permutation methods to adjust for the standard error of within-participant correlation of the teeth at each time point. Of note, neither method of standard error computation accounts for within-participant correlation over time, which could artificially lower the standard errors and thus requires a cautious interpretation of these tests.

Next, we cross-tabulated changes in mean FRI category between adjacent time points for each tooth group at the person- and tooth-levels and plotted the results in grouped bar plots to facilitate visualization of the trends over time. We calculated 95% confidence intervals (CIs) to be used as error bars on the grouped bar plots. For the person-level mean FRI, a CI based on inversion of a binomial score test was calculated and plotted for cross-tabulation cells containing at least 10 participants. At the tooth level, we accounted for intra-participant clustering by calculating CIs using generalized linear models for binary outcomes with a logit link function, and fitting the models using generalized estimating equations (PROC GENMOD, SAS version 9.4, SAS Institute Inc., Cary, NC).

For each participant included in the cross-tabulation tables and accompanying figures, we calculated the difference between baseline and follow-up mean FRI scores using the difference between the actual mean FRI **scores**—not the mean FRI **categories**. Then, we carried out Wilcoxon signed-rank tests stratified by baseline mean FRI category to determine whether the probability of having a lower follow-up mean FRI score was significantly greater than the probability of having a higher follow-up mean FRI score, indicating a significant decline in mean fluorosis severity. At the tooth-level, we utilized the clusrank package in R to calculate a version of this test which accounted for intra-participant correlation (17, 18).

A significance level of 0.05 was used for all tests, and non-directional *p*-values were used for all tests except the Wilcoxon signed-rank tests because we were testing for a decrease in mean FRI score.

## Results

Total counts of IFS participants with dental exams at ages 9, 13, 17, and 23 were 629, 549, 464, and 342, respectively. The mean (standard deviation) of actual ages (in years) at each dental exam was 9.3 (0.7), 13.5 (0.6), 17.8 (0.7), and 23.5 (0.6). The sample of examined participants had slightly more females at all exams (50.6–56.7% female at each exam), was primarily non-Hispanic white (94.8–95.9%), and in 2007 about 68% of participant households had income ≥$60,000 and 50.6–56.7% of mothers had at least a four-year college degree. Mean **person-level** pairwise inter-examiner reliability kappa statistics for the 4 mean FRI score categories were 0.50, 0.34, 0.25, and 0.15 for ages 9, 13, 17, and 23, respectively. Mean **tooth-level** pairwise inter-examiner reliability kappa statistics for the 4 mean FRI score categories were 0.48, 0.33, 0.29, and 0.16 for ages 9, 13, 17, and 23, respectively.

For all participants (and zones) examined at all four time points, we summarized the mean FRI score categories at each exam in Table 2. In this table, we observed steadily higher percentages of participants in the mean FRI of 0 category at later ages for all tooth groups, although this trend appeared to slow down between ages 17 and 23 years. The other, higher mean FRI categories generally had lower percentages of patients at later ages, indicating a trend of lower mean FRI category at later ages for all tooth groups. This trend was supported by the gamma statistics, which were negative and significantly less than zero (*p* < 0.0001) for all tooth groups.

**Table 2:** Average person-level FRI scores [count (%) of subjects].

Tooth Type (Gamma, *p*-value)*	Mean Person-Level FRI Score Category	Age 9 (*n* = 282) (Permanent Teeth Only)	Age 13 (*n* = 282)	Age 17 (*n* = 282)	Age 23 (*n* = 282)
Early-Erupting Teeth (−0.35, < 0.0001)	0	84 (29.8)	127 (45.0)	164 (58.2)	173 (61.4)
	0.01–0.50	146 (51.8)	127 (45.0)	103 (36.5)	100 (35.5)
	0.51–1.00	35 (12.4)	23 (8.2)	12 (4.3)	5 (1.8)
	1.01–3.00	17 (6.0)	5 (1.8)	3 (1.1)	4 (1.4)
Late-Erupting Teeth (−0.34, < 0.0001)			(*n* = 291)	(*n* = 291)	(*n* = 291)
	0	–	114 (39.2)	167 (57.4)	192 (66.0)
	0.01–0.50	–	130 (44.7)	106 (36.4)	86 (29.6)
	0.51–1.00	–	35 (12.0)	10 (3.4)	8 (2.8)
	1.01–3.00	–	12 (4.1)	8 (2.8)	5 (1.7)
Maxillary Incisors (−0.35, < 0.0001)		(*n* = 279)	(*n* = 279)	(*n* = 279)	(*n* = 279)
	0	100 (35.8)	141 (50.5)	181 (64.9)	192 (68.8)
	0.01–0.50	87 (31.2)	85 (30.5)	59 (21.2)	59 (21.2)
	0.51–1.00	59 (21.2)	37 (13.3)	33 (11.8)	23 (8.2)
	1.01–3.00	33 (11.8)	16 (5.7)	6 (2.2)	5 (1.8)

* Gamma statistic for association between age and fluorosis severity. *p*-value was calculated under the assumption that the sample size was sufficiently large for (gamma statistic/asymptotic standard error) to approximately follow a standard normal distribution.

Next, we summarized the mean tooth-level FRI score categories for the zones scored at all four exams in Table 3. Compared to the participant-level results in Table 2, there were relatively higher percentages of teeth in the 0 FRI category. Although many participants had some fluorosis, most teeth did not have any fluorosis. After accounting for the higher percentages of teeth in the 0 category, the trends in Table 3 are similar to the trends in Table 2. Namely, there were significantly higher percentages of teeth in the 0 category and lower percentages of teeth in all other categories at later ages. One difference in trends between Tables 2, 3 was the more substantial leveling-off of the decline in fluorosis category observed from ages 17 to 23 for the late-erupting teeth in the 0 and 0.51–1.00 mean FRI categories.

**Table 3:** Average tooth-level FRI scores [count (%) of teeth].

Tooth Type (Gamma, *p*-value)*	Average Tooth-Level FRI Score Category	Age 9 (*n* = 3,269) (Permanent Teeth Only)	Age 13 (*n* = 3,269)	Age 17 (*n* = 3,269)	Age 23 (*n* = 3,269)
Early-Erupting Teeth (−0.31, < 0.0001)	0	2,249 (68.8)	2,577 (78.8)	2,791 (85.4)	2,847 (87.1)
	0.01–0.50	428 (13.1)	338 (10.3)	252 (7.7)	239 (7.3)
	0.51–1.00	323 (9.9)	220 (6.7)	152 (4.7)	133 (4.1)
	1.01–3.00	269 (8.2)	134 (4.1)	74 (2.3)	50 (1.5)
Late-Erupting Teeth (−0.28, < 0.0001)			(*n* = 4,251)	(*n* = 4,251)	(*n* = 4,251)
	0	–	2,871 (67.5)	3,368 (79.2)	3,543 (83.4)
	0.01–0.50	–	798 (18.8)	608 (14.3)	501 (11.8)
	0.51–1.00	–	402 (9.5)	160 (3.8)	131 (3.1)
	1.01–3.00	–	180 (4.2)	115 (2.7)	76 (1.8)
Maxillary Incisors (−0.32, < 0.0001)		(*n* = 1,068) (Permanent Teeth Only)	(*n* = 1,068)	(*n* = 1,068)	(*n* = 1,068)
	0	547 (51.2)	683 (64.0)	792 (74.2)	831 (77.8)
	0.01–0.50	216 (20.2)	201 (18.8)	151 (14.1)	143 (13.4)
	0.51–1.00	179 (16.8)	121 (11.3)	98 (9.2)	75 (7.0)
	1.01–3.00	126 (11.8)	63 (5.9)	27 (2.5)	19 (1.8)

* Gamma statistic for association between age and fluorosis severity. *p*-value was calculated using a permutation test method to account for clustering of teeth within participants. In addition, *p*-value was calculated under the assumption that the sample size was sufficiently large for (gamma statistic/asymptotic standard error) to approximately follow a standard normal distribution.

**Figure 1:**
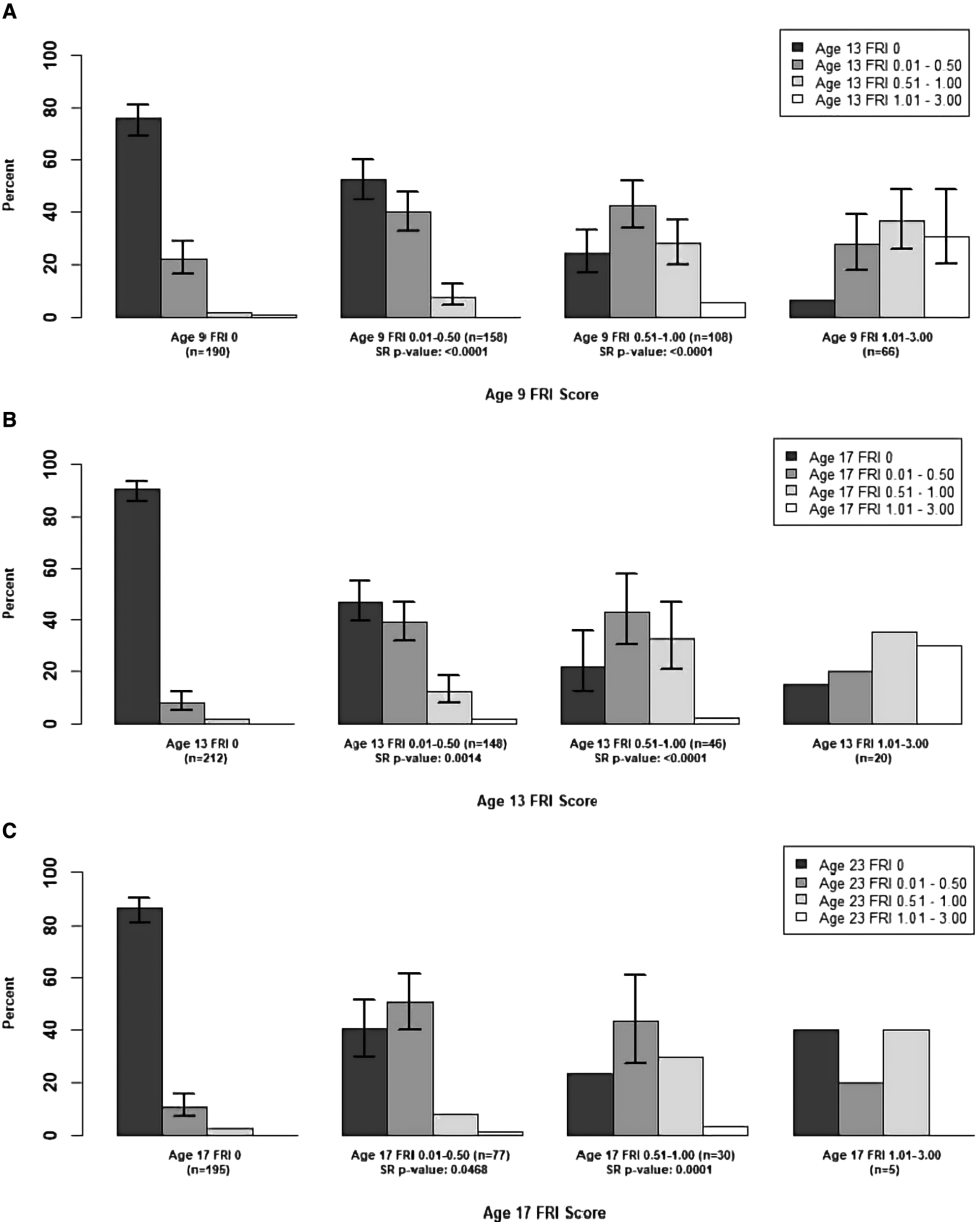
Summary of Transitions for Person-level Average FRI Scores at Each Exam for the Maxillary Incisors. Error bars represent 95% CIs based on the score test for binomial proportions. Confidence intervals provided for bars representing ≥10 participants. For the middle two baseline score categories, one-sided Wilcoxon signed-rank (SR) p-values are provided for testing whether there was a higher probability of decreasing mean FRI score from one time point to the next. Actual differences in the mean FRI score between baseline and follow-up were used for calculation of this test statistic, instead of differences between categories. (**A**) Mean Person-Level FRI Score for the Maxillary Incisors, 9-13 years. (**B**) Mean Person-Level FRI Score for the Maxillary Incisors, 13-17 years. (**C**) Mean Person-Level FRI Score for the Maxillary Incisors, 17-23 years.

**Figure 2:**
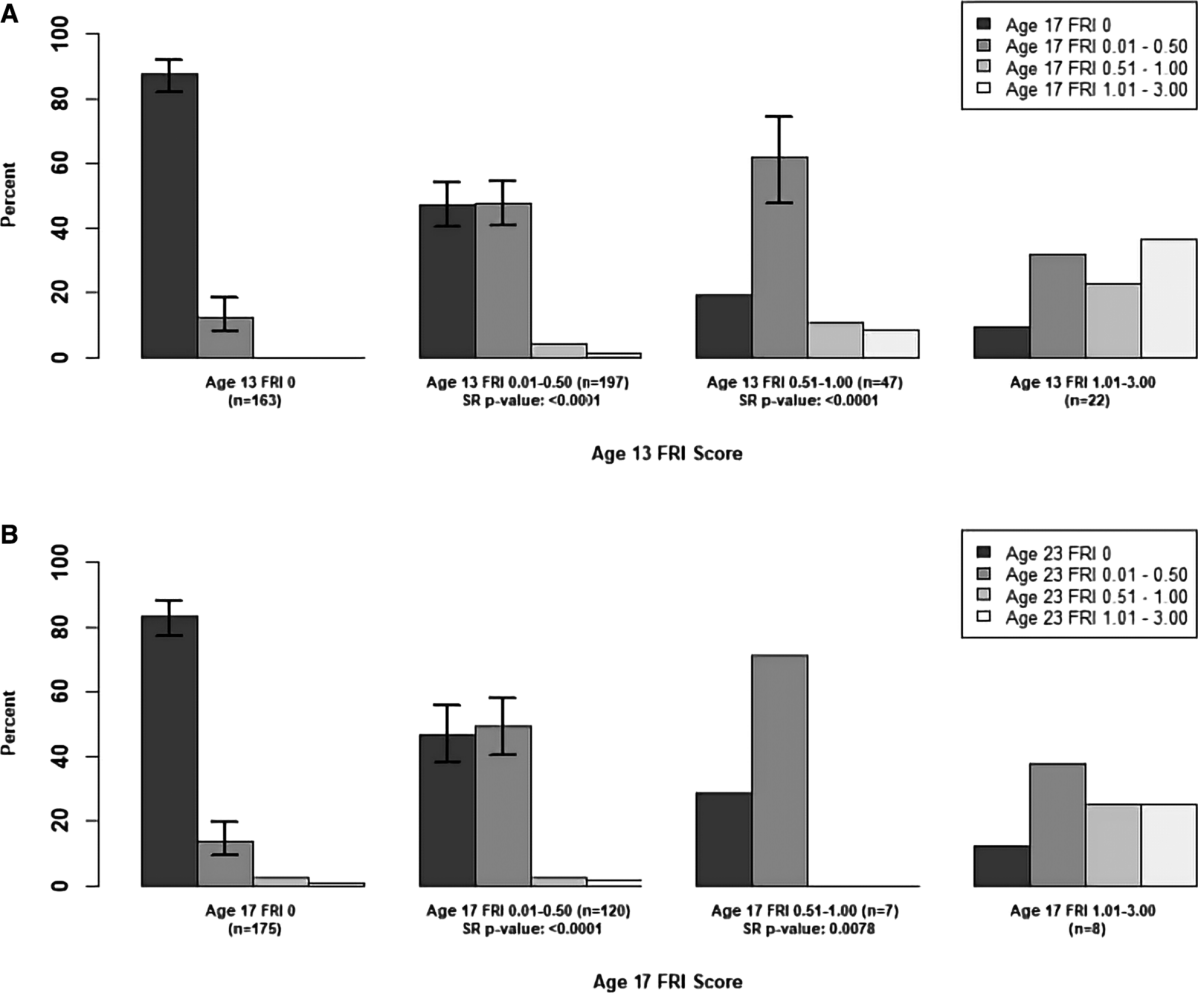
Summary of Transitions for Person-level Average FRI Scores at Each Exam for the Late-Erupting Teeth. Error bars represent 95% confidence intervals based on the score test for binomial proportions. Confidence intervals were only provided for bars which represent at least 10 participants. For the middle two baseline score categories, one-sided Wilcoxon signed-rank (SR) *p*-values are provided for testing whether there was a higher probability of decreasing mean FRI score from one time point to the next. Actual differences in the mean FRI score between baseline and follow-up were used for calculation of this test statistic, instead of differences between categories. (**A**) Mean Person-Level FRI Score for the Late-Erupting Teeth, 13-17 years. (**B**) Mean Person-Level FRI Score for the Late-Erupting Teeth, 17-23 years.

**Figure 3:**
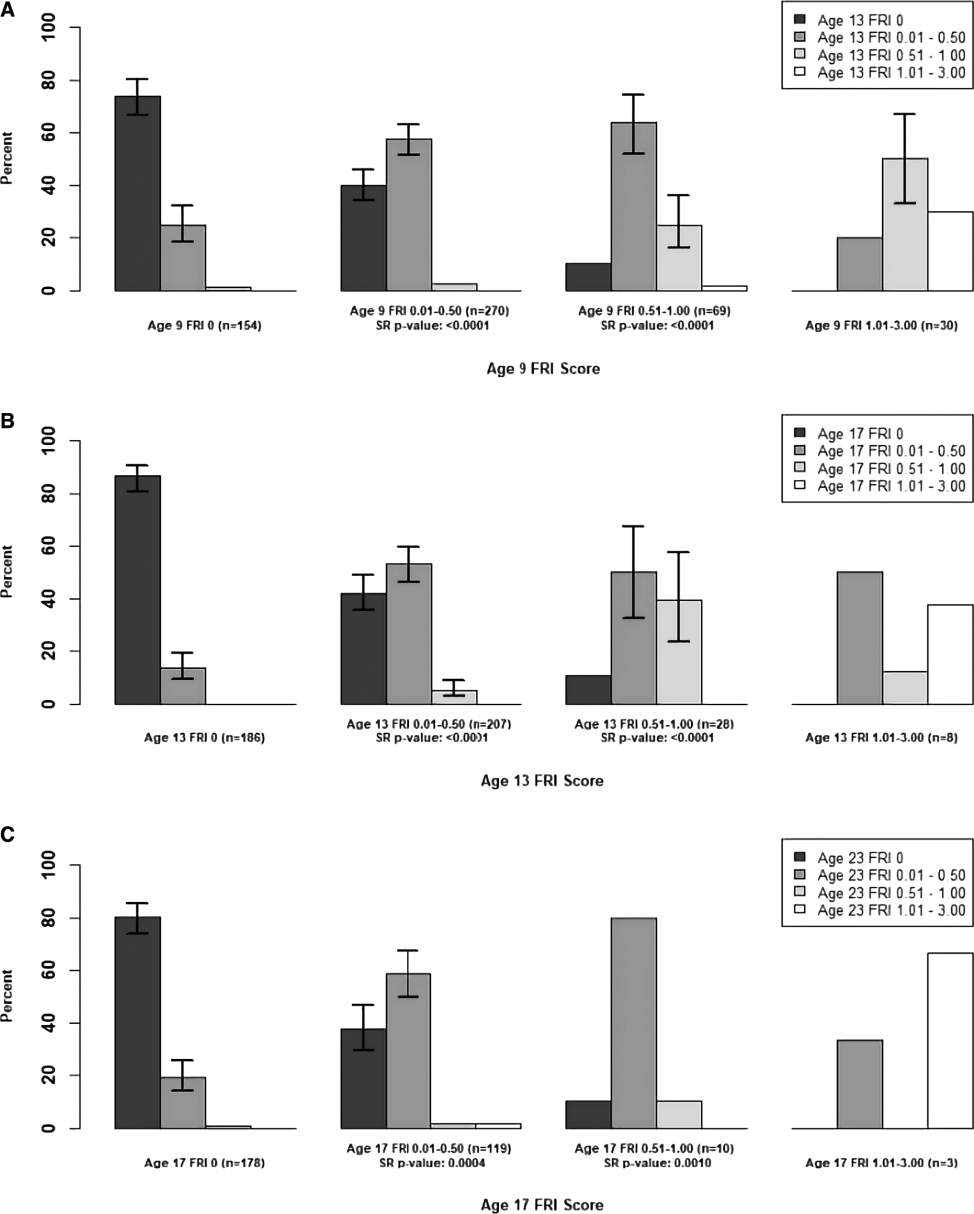
Summary of Transitions for Person-level Average FRI Scores at Each Exam for the Early-Erupting Teeth. Error bars represent 95% confidence intervals based on the score test for binomial proportions. Confidence intervals were only provided for bars which represent at least 10 participants. For the middle two baseline score categories, one-sided Wilcoxon signed-rank (SR) p-values are provided for testing whether there was a higher probability of decreasing mean FRI score from one time point to the next. Actual differences in the mean FRI score between baseline and follow-up were used for calculation of this test statistic, instead of differences between categories. (**A**) Mean Person-Level FRI Score for the Early-Erupting Teeth, 9-13 years. (**B**) Mean Person-Level FRI Score for the Early-Erupting Teeth, 13-17 years. (**C**) Mean Person-Level FRI Score for the Early-Erupting Teeth, 17-23 years.

In Figure 1, we examined the changes in mean **person-level** FRI scores between adjacent time points for the maxillary incisors, and in Figures 2, 3, we examined these changes for the late- and early-erupting teeth, respectively. Starting with the Wilcoxon Signed-Rank test results, we point out that all Wilcoxon Signed-Rank test statistics were negative, indicating that participants were more likely to experience a decrease in mean FRI from baseline to follow-up, relative to an increase in mean FRI. All *p*-values were statistically significant at the 0.05 level, but some larger *p*-values were observed from ages 17–23. This may be due to smaller sample sizes in these baseline categories at age 17.

For the participants with a baseline mean FRI of 0 in Figures 1–3, most participants had a mean FRI of 0 at follow-up. The increase in fluorosis severity observed in a relatively small number of participants likely was due to examiner variability. The highest percentages of participants with baseline FRI of 0 and follow-up mean FRI of 0.01–0.50 was observed from ages 9 to 13.

Participants in the mean FRI 0.01–0.50 group at baseline were split approximately evenly between the 0 and the 0.01–0.50 category at follow-up. This was consistent across most tooth groups and age pairs, and most confidence intervals for the percentage of participants in each of the lower two follow-up categories overlap. One exception to this was the early-erupting teeth from ages 9–13 and 17–23 where there were significantly fewer participants who decreased from mean FRI 0.01–0.50 to 0 relative to those who stayed the same. Relatively few participants with a baseline mean FRI of 0.01–0.50 transitioned into the two highest mean FRI categories.

For the baseline 0.51–1.00 category participants in Figures 1–3 at follow-up, the highest percentage of participants were consistently in the FRI 0.01–0.50 category. This indicates a decline in fluorosis severity. However, many of the follow-up groups for these participants do not have sufficient data to calculate 95% confidence intervals, especially for later pairs of time points. Finally, the percentage estimates in the highest baseline category (mean FRI of 1.01–3.00) are based on relatively few participants, which limits our ability to draw firm conclusions.

**Figure 4:**
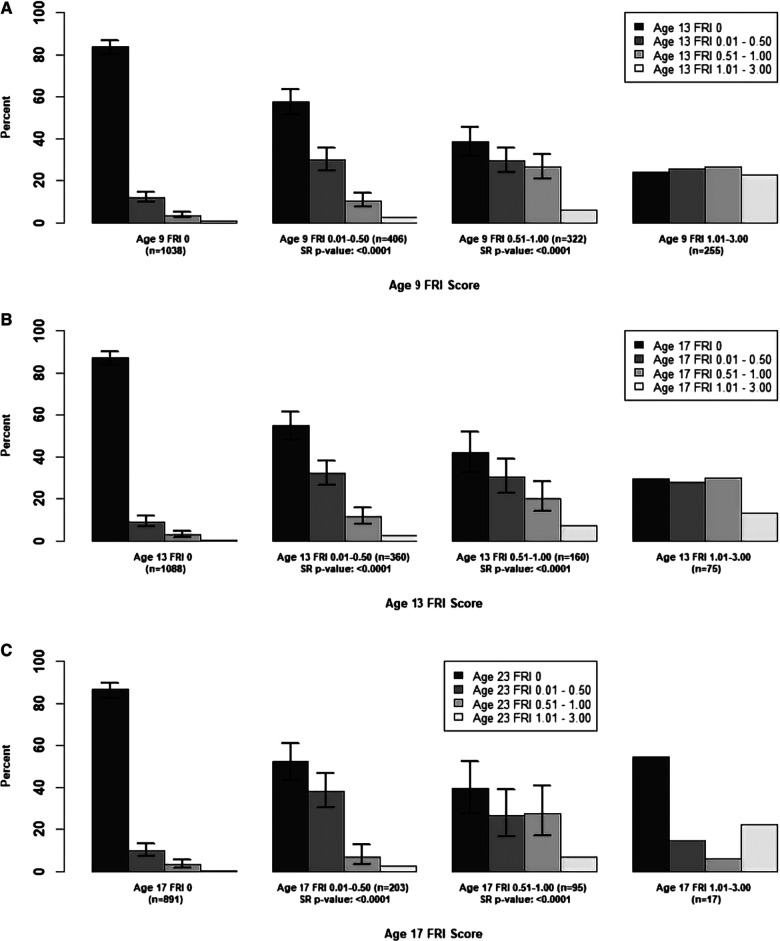
Summary of Transitions for Tooth-level Average FRI Scores at Each Exam for the Maxillary Incisors. Error bars represent 95% CIs based on binomial generalized estimating equations. For the middle two baseline categories, one-sided Wilcoxon signed-rank (SR) p-values are provided for testing whether there was a higher probability of decreasing mean FRI score from one time point to the next. Actual differences in the mean FRI score between baseline and follow-up were used for calculation of this test statistic, instead of differences between categories. Permutation methods have been used to account for intra-participant clustering of teeth when calculating these p-values. (**A**) Mean Tooth-Level FRI Score for the Maxillary Incisors, 9-13 years. (**B**) Mean Tooth-Level FRI Score for the Maxillary Incisors, 13-17 years. (**C**) Mean Tooth-Level FRI Score for the Maxillary Incisors, 17-23 years.

**Figure 5:**
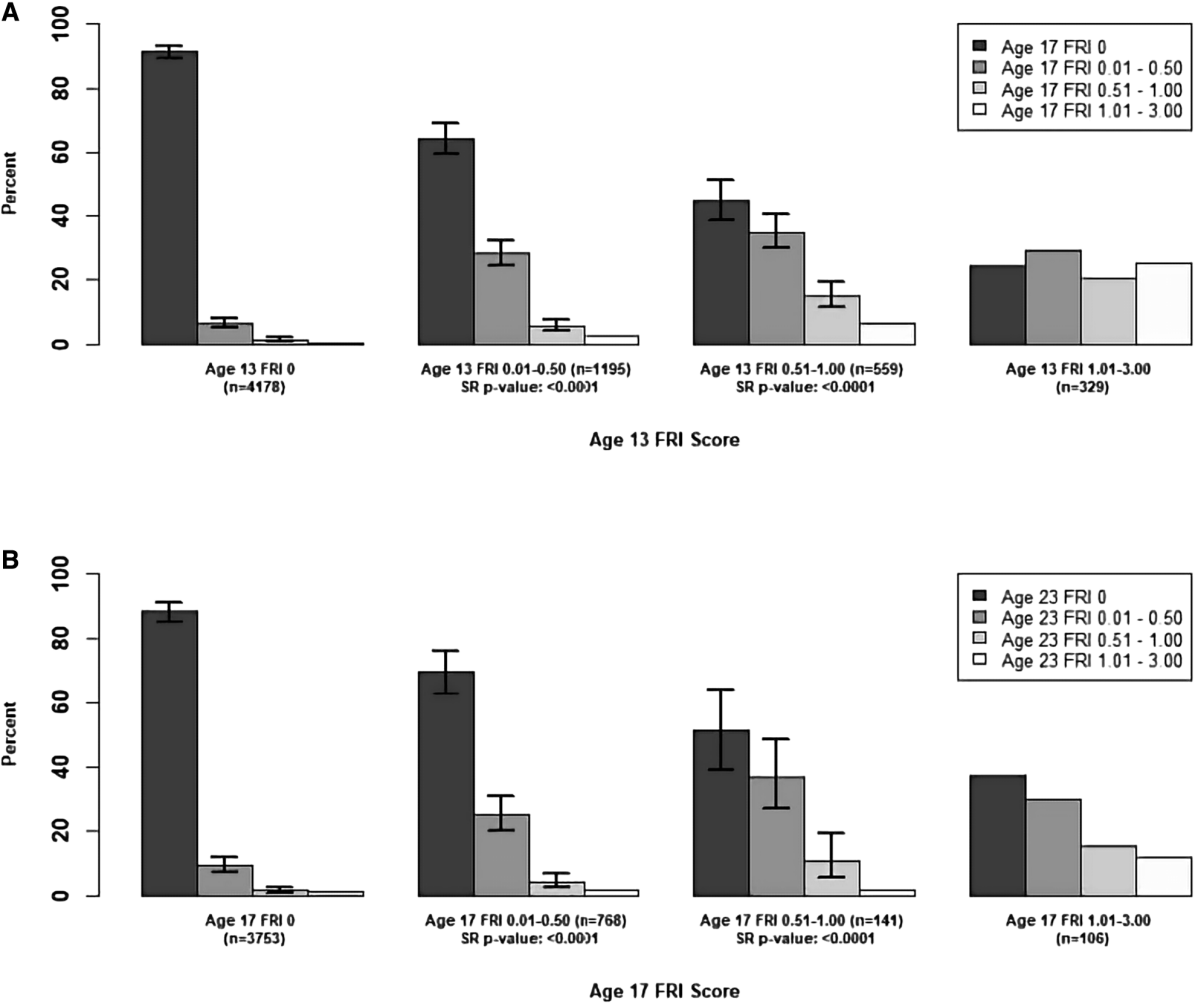
Summary of Transitions for Tooth-level Average FRI Scores at Each Exam for Late-Erupting Teeth. Error bars represent 95% confidence intervals based on binomial generalized estimating equations which account for intra-participant correlation. For the middle two baseline score categories, one-sided Wilcoxon signed-rank (SR) p-values are provided for testing whether there was a higher probability of decreasing mean FRI score from one time point to the next. Actual differences in the mean FRI score between baseline and follow-up were used for calculation of this test statistic, instead of differences between categories. Permutation methods have been used to account for intra-participant clustering of teeth when calculating these *p*-values. (**A**) Mean Tooth-Level FRI Score for the Late-Erupting Teeth, 13-17 years. (**B**) Mean Tooth-Level FRI Score for the Late-Erupting Teeth, 17-23 years.

**Figure 6:**
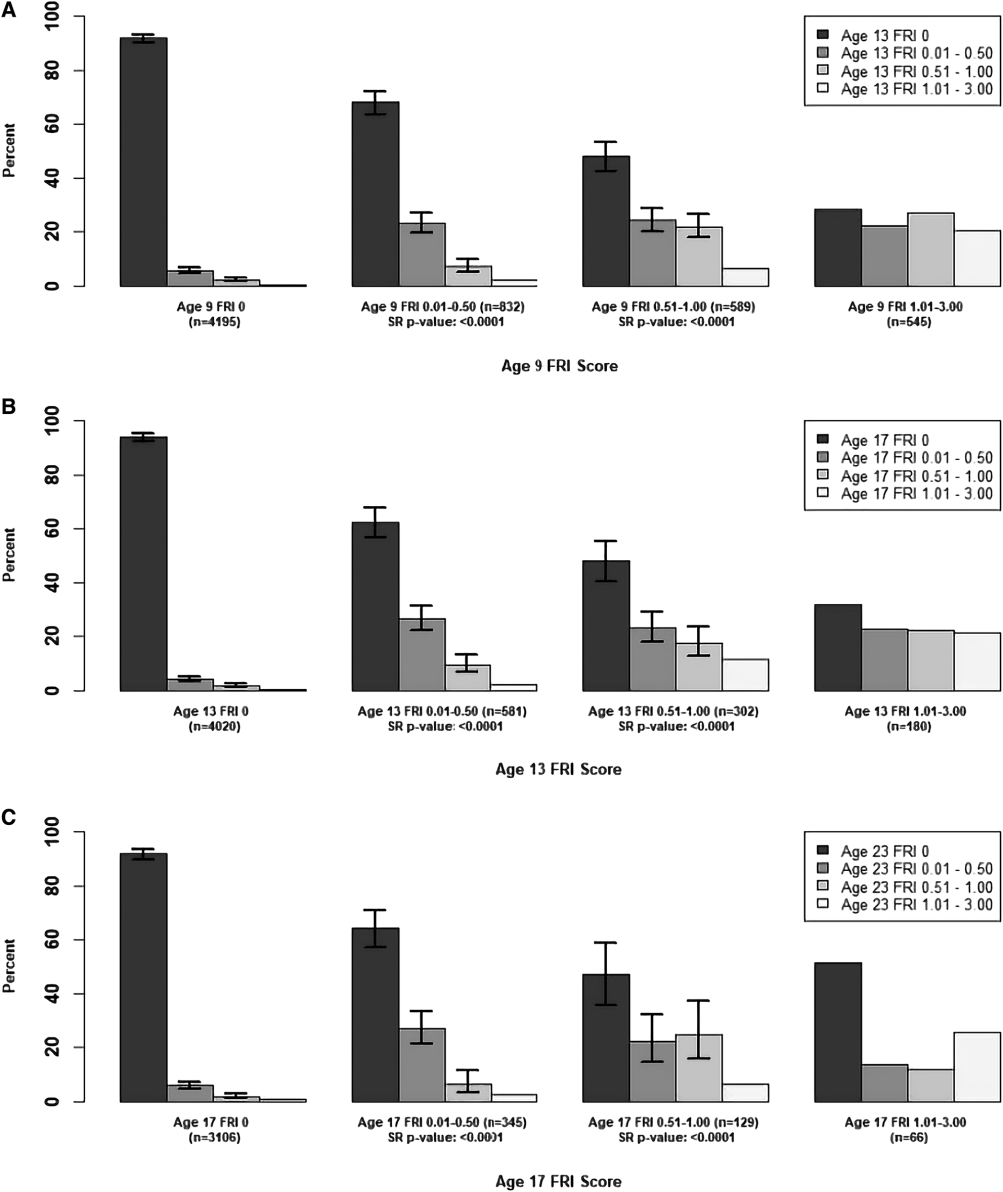
Summary of Transitions for Person-level Average FRI Scores at Each Exam for the Early-Erupting Teeth. Error bars represent 95% confidence intervals based on the score test for binomial proportions. Confidence intervals were only provided for bars which represent at least 10 participants. For the middle two baseline score categories, one-sided Wilcoxon signed-rank (SR) p-values are provided for testing whether there was a higher probability of decreasing mean FRI score from one time point to the next. Actual differences in the mean FRI score between baseline and follow-up were used for calculation of this test statistic, instead of differences between categories. Permutation methods have been used to account for intra-participant clustering of teeth when calculating these *p*-values. (**A**) Mean Tooth-Level FRI Score for the Early-Erupting Teeth, 9-13 years. (**B**) Mean Tooth-Level FRI Score for the Early-Erupting Teeth, 13-17 years. (**C**) Mean Tooth-Level FRI Score for the Early-Erupting Teeth, 17-23 years.

In Figures 4–6 we examined transitions in **tooth-level** mean FRI scores at pairs of examinations. The Wilcoxon signed-rank test statistics were all negative and had highly significant *p*-values for all baseline groups. Examining the trends over time as presented in the bar plots, we start with the teeth which had a baseline mean FRI of zero. These teeth were primarily scored as 0 at follow-up, with relatively few teeth falling into the mean FRI 0.01–0.50 category. This difference (in steady vs. declining mean FRI scores) was statistically significant for all tooth types and age pairs.

For the teeth with a baseline mean FRI of 0.01–0.50, the most common follow-up category was mean FRI of 0, with the second highest percentage of teeth remaining in the 0.01–0.50 category at follow-up, the third highest percentage of teeth in the 0.50–1.00 category at follow-up, and the fewest participants in the 1.01–3.00 category at follow-up. The follow-up percentages for the three lowest mean FRI categories generally had non-overlapping 95% confidence intervals, indicating statistically significant differences. One exception was for the maxillary incisors from ages 17–23, where the lowest two mean FRI categories had overlapping confidence intervals for the percentage of participants in each follow-up category.

For teeth in the baseline 0.51–1.00 category, the most common follow-up category was FRI of 0, indicating a decline in general fluorosis severity. From ages 9–13, the percentage of teeth with a baseline mean FRI of 0.51–1.00 who were in the follow-up mean FRI categories of 0.01–0.50 and 0.51–1.00 were relatively similar, with overlapping confidence intervals. From ages 13–17, there were generally a higher percentage of teeth in the mean FRI 0.01–0.50 follow-up group compared to those who remained in the mean FRI 0.51–1.00 follow-up group, with significantly higher percentages of teeth in the 0.51–1.00 group for the late-erupting teeth. From ages 17–23, there were significantly higher percentages of teeth in the lower follow-up categories (FRI of 0 and mean FRI of 0.01–0.50) relative to those who remained in the 0.51–1.00 mean FRI category for the late-erupting teeth. However, we observed overlapping CIs for these groups for the early-erupting teeth and maxillary incisors, which may indicate a leveling-off in the decline in fluorosis severity for the early-erupting teeth and maxillary incisors.

There is a relatively small number of teeth in the mean FRI 1.01–3.00 baseline category. Here, we see similar numbers of teeth in each follow-up category from ages 9–13 and 13–17 for all tooth groups.

In addition to the trends of decline for the mean FRI scores at the person- and tooth-levels, there was a trend of decline in the five-category generalized fluorosis measure, as seen in Table 4. The percentages of participants in the “No Fluorosis” and “Any Questionable” categories were higher at later time points, and the percentages of participants in all other categories, including both generalized fluorosis categories, were lower at later time points. A substantial percentage of participants were included in one of the two generalized fluorosis categories at the first time point (41.5% of participants for the early-erupting teeth at age 9, 34% of participants at age 13 for the late-erupting teeth, and 54.1% of participants for the maxillary incisors at age 9), which indicated that there was a sizable amount of generalized fluorosis present in the IFS cohort at early time points. We observed a much lower percentage of participants in either of the generalized fluorosis categories at age 23 (8.9%, 16.1%, and 27.2% for the early-erupting, late-erupting teeth, and maxillary incisors, respectively). This declining trend in the percentage of participants with generalized fluorosis was reflected in the negative and statistically significant gamma statistics observed for each tooth group.

**Table 4:** Summary of five-category generalized fluorosis outcomes at the four dental exams. See Table 1 for definitions of each category.

Tooth Type (Gamma statistic, *p*-value)*	Generalized Fluorosis Category	Age 9 (Permanent Teeth Only) (*n* = 282)	Age 13 (*n* = 282)	Age 17 (*n* = 282)	Age 23 (*n* = 282)
Early- Erupting (−0.38, < 0.0001)	No Fluorosis	84 (29.8)	127 (45.0)	164 (58.2)	173 (61.4)
	Questionable** (not meeting Positive threshold)	35 (12.4)	73 (25.9)	66 (23.4)	63 (22.3)
	Any Positive	46 (16.3)	29 (10.3)	18 (6.4)	21 (7.5)
	Generalized Questionable**	40 (14.2)	41 (14.5)	27 (9.6)	21 (7.5)
	Generalized Positive	77 (27.3)	12 (4.3)	7 (2.5)	4 (1.4)
Late-Erupting (−0.31, < 0.0001)			(*n* = 291)	(*n* = 291)	(*n* = 291)
	No Fluorosis	–	114 (39.2)	167 (57.4)	192 (66.0)
	Questionable** (not meeting Positive threshold)	–	71 (24.4)	59 (20.3)	51 (17.5)
	Any Positive	–	7 (2.4)	8 (2.8)	1 (0.3)
	Generalized Questionable**	–	74 (25.4)	48 (16.5)	41 (14.1)
	Generalized Positive	–	25 (8.6)	9 (3.1)	6 (2.1)
Maxillary Incisors (−0.33, < 0.0001)		(Permanent Teeth Only) (*n* = 279)	(*n* = 279)	(*n* = 279)	(*n* = 279)
	No Fluorosis	100 (35.8)	141 (50.5)	181 (64.9)	192 (68.8)
	Questionable** (not meeting Positive threshold)	28 (10.0)	23 (8.2)	16 (5.7)	11 (3.9)
	Any Positive	0 (0.0)	1 (0.4)	0 (0.0)	0 (0.0)
	Generalized Questionable**	64 (22.9)	74 (26.5)	55 (19.7)	53 (19.0)
	Generalized Positive	87 (31.2)	40 (14.3)	27 (9.7)	23 (8.2)

* Gamma statistic for association between age and fluorosis severity. *p*-value was calculated under the assumption that the sample size was sufficiently large for (gamma statistic/asymptotic standard error) to approximately follow a standard normal distribution.

** Note that the FRI's “questionable” category includes many zones with fluorosis affecting less than 50% of the zone, as well as some zones that do not have fluorosis.

In addition to the main analyses described above, we also assessed the effects of the reported use of tooth bleaching and whitening products on the observed decrease in fluorosis severity. We performed a sensitivity analysis to compare changes in mean fluorosis severity among those who reported using bleaching/whitening products and those who reported no such usage. As described in the Supplementary Appendix, we used non-parametric Wilcoxon rank-sum tests to assess differences between mean baseline and follow-up FRI scores among the three tooth groups for those who reported use of bleaching/whitening products at least once during the study period and those who reported no use. At the tooth-level, rank-sum tests which account for intra-participant clustering were utilized (17, 19).

At the person-level, there were no clear differences based on bleaching/whitening product use, and no meaningful or statistically significant differences between these groups. At the tooth-level, there were generally very few differences between bleaching groups. However, there was a statistically significant (*p* = 0.011) difference in the change in mean FRI scores for the late-erupting teeth from 13 to 17 among those in mean baseline FRI category 0.51–1.00. Here, participants who used bleaching/whitening products had a greater mean change (0.63) than those who didn't (0.42). These findings must be interpreted with caution due to the multiple comparisons used (16 hypothesis tests each for person- and tooth-level comparisons). Please see the Supplementary Appendix for additional sensitivity analysis results.

## Discussion

Based on these results and those previously published (12), data from the IFS have demonstrated that fluorosis becomes less evident over time. This was found for both the more severe fluorosis lesions observed for a participant or tooth (12), as well as here as a reduction in the amount of generalized fluorosis seen throughout the mouth or across the zones of a tooth. We were concerned that this similarity between the trend in the most severe fluorosis lesions and mean FRI score was due to a lack of generalized fluorosis in our cohort. However, after utilizing an additional five-category measure of carefully defined generalized fluorosis measures, we also observed a trend of less severe generalized fluorosis and less generalized fluorosis over time.

These findings add to the results from the limited number of cohort studies which examined changes in fluorosis severity over time. Most of these studies demonstrated a decline in fluorosis severity (9–12), while one relatively small study did not (20). While most studies measured fluorosis severity based on the most severe level of fluorosis observed for a participant and examined changes in this measure over time, we have examined two measures of generalized fluorosis severity, which provides more information about the status of fluorosis throughout the mouth, and could be more important from an esthetic perspective.

Limitations of this analysis include the racial and geographic homogeneity and relatively high socioeconomic status of the IFS cohort. In addition, there were low numbers of severe fluorosis cases observed and substantial loss to follow-up over the 23-year period of the study. The five-category generalized fluorosis outcome variable was empirical, not validated and not based on previous literature, so that these results should not be considered definitive, but they do demonstrate the overall trend toward fluorosis becoming less evident over time. The effect of tooth whitening products on the observed trend of declining fluorosis severity was another potential limitation; however, there was limited evidence of a difference in the decline in fluorosis severity between participants who reported using none vs. any bleaching products. There was high inter-examiner variability—especially at later ages—which appears to be an inherent problem in scoring fluorosis (7), but nonetheless is a concern in any clinical study. Also, the FRI was designed for case-control studies and was chosen to relate fluoride intakes to fluorosis to specific tooth zones; thus, it may not have been the ideal index for assessing changes over time. Finally, the “questionable” category may not have consistently measured the presence of fluorosis; however, as defined by Pendrys (14), this category was indicated when fluorosis did not reach the threshold of at least 50% of a tooth zone affected and was not meant to record areas not affected by fluorosis.

On the other hand, the strengths of this study include assessment of fluorosis severity for the IFS cohort at four time points over the course of 14 years, differential diagnosis of fluorosis, enamel hypoplasia and opacities, and assessing changes in generalized fluorosis as opposed to changes in the most severe fluorosis sites.

Overall, these results showed that generalized fluorosis severity became less evident throughout adolescence and early adulthood. Other cohort studies have also shown a reduction in severity of the most severe case of fluorosis in the mouth or over a tooth group. Taken together, these data suggest that policymakers and those tasked with quantifying the prevalence and severity of fluorosis in a population should account for the fact that fluorosis severity measured in childhood or early adolescence probably provides an over-estimate of both generalized fluorosis presentation and the most severe fluorosis in adult populations. Also, clinicians should consider this information prior to unnecessarily removing mild enamel fluorosis for esthetic purposes in young patients.

## Data Availability

The datasets presented in this article are not readily available. Interested parties can request the data and inquiries will be reviewed for meeting all institutional and other scientific requirements for sharing the data. Requests to access the datasets should be directed to steven-levy@uiowa.edu.
