# Supplementary material for: Generalized permanent dentition fluorosis severity becomes less evident over time among a birth cohort
Source: Front Oral Health. 2023 Jun 30;4:1198167. doi: 10.3389/froh.2023.1198167 (PMC10348053; doi:10.3389/froh.2023.1198167)
Supplement: Supplementary file 1 [file Datasheet1.docx]

Appendix for Frontiers Longitudinal Fluorosis Paper 6/1/23

Contents

- Bleaching Sensitivity Analysis
  - Introduction
  - Appendix Table 1
  - Appendix Table 2
  - Appendix Table 3
  - Appendix Table 4

**Appendix**

**Bleaching Sensitivity Analysis - Introduction**

In order to check for a difference in trends between the participants who did not report using any bleaching products (n = 418) and those who reported using bleaching products in at least one follow-up questionnaire (n = 189), we used Wilcoxon rank-sum tests (non-parametric analogs of the two-sample t-test). These tests assess whether the distributions of differences in mean FRI from baseline to follow-up (stratified by baseline mean FRI category) were significantly different between the bleaching and non-bleaching groups.

The Wilcoxon rank-sum test provides us with a one-sided or two-sided p-value for whether or not the distributions of two independent (unpaired) groups are different. (In this case, the two groups we examine are participants who used any bleaching products and participants who did not use any bleaching products, and the variable we are examining is the difference in mean FRI score between baseline and follow-up.) However, unlike a t-test, the test statistic for a rank-sum test does not provide us with an estimate of the direction or easily interpretable estimate of the magnitude of the difference between the two groups. Therefore, we also provide summary statistics to help understand the differences between the groups on the following pages.

Appendix Table 1 – Sample size and mean (standard deviation) of the differences in mean FRI from baseline to follow-up at the person level for each tooth group and pair of adjacent ages, stratified by whether participants reported using any bleaching products or not. Cells highlighted in light grey represent cases where the any bleaching group had a slightly more negative mean difference in mean FRI from baseline to follow-up compared to the no bleaching group (difference of < 0.02). Dark grey highlighting is used to represent cases where the negative mean difference was greater than 0.02 in magnitude.

| Tooth Group | Mean FRI Cat. | Summary Statistics | Adjacent Ages | | | | | |
| --- | --- | --- | --- | --- | --- | --- | --- | --- |
|  |  |  | 9 – 13 | | 13 – 17 | | 17 – 23 | |
| Any Bleaching? | | | No | Yes | No | Yes | No | Yes |
| Early-Erupting | 0.01– 0.50 | N | 188 | 82 | 128 | 79 | 67 | 52 |
|  |  | Mean  (SD) | -0.08 (0.16) | -0.09 (0.14) | -0.04 (0.18) | -0.05 (0.18) | -0.03 (0.22) | -0.03 (0.22) |
|  | 0.51– 1.00 | N | 35 | 34 | 17 | 11 | 7 | 3 |
|  |  | Mean  (SD) | -0.37 (0.31) | -0.41 (0.25) | -0.31 (0.33) | -0.35 (0.22) | -0.66 (0.15) | -0.43 (0.13) |
| Late-Erupting | 0.01– 0.50 | N | - | - | 126 | 71 | 70 | 50 |
|  |  | Mean  (SD) | - | - | -0.06 (0.17) | -0.07 (0.25) | -0.04 (0.25) | -0.04 (0.21) |
|  | 0.51– 1.00 | N | - | - | 31 | 16 | 5 | 2 |
|  |  | Mean  (SD) | - | - | -0.33 (0.39) | -0.41 (0.19) | -0.60 (0.08) | -0.73 (0.32) |
| Maxillary Incisors | 0.01– 0.50 | N | 106 | 52 | 91 | 57 | 45 | 32 |
|  |  | Mean  (SD) | -0.07 (0.22) | -0.12 (0.22) | -0.05 (0.28) | -0.03 (0.32) | -0.06 (0.25) | -0.01 (0.28) |
|  | 0.51– 1.00 | N | 71 | 37 | 26 | 20 | 17 | 13 |
|  |  | Mean  (SD) | -0.42 (0.34) | -0.32 (0.40) | -0.35 (0.34) | -0.41 (0.35) | -0.32 (0.58) | -0.29 (0.39) |

Appendix Table 2 – p-values for two-sided Wilcoxon rank-sum statistics testing for a difference in the distribution of baseline to follow-up mean person-level FRI differences between participants with and without reporting use of any bleaching products.

| Tooth Group | | Early-Erupting | | Late-Erupting | | Maxillary Incisors | |
| --- | --- | --- | --- | --- | --- | --- | --- |
| Baseline Mean FRI Category | | 0.01 – 0.50 | 0.51 – 1.00 | 0.01 – 0.50 | 0.51 – 1.00 | 0.01 – 0.50 | 0.51 – 1.00 |
| Adjacent Ages | 9 – 13 | 0.80 | 0.74 | - | - | 0.12 | 0.32 |
|  | 13 – 17 | 0.38 | 0.68 | 0.59 | 0.94 | 0.98 | 0.49 |
|  | 17 – 23 | 0.66 | 0.10 | 0.92 | 0.85 | 0.41 | 0.53 |

Appendix Table 3 – Sample size and mean (standard deviation) of the differences in mean FRI from baseline to follow-up at the tooth level for each tooth group and pair of adjacent ages, stratified by whether participants reported using any bleaching products or not.

Cells highlighted in light grey represent cases where the any bleaching group had a slightly more negative mean difference in mean FRI from baseline to follow-up compared to the no bleaching group (difference of < 0.02). Dark grey highlighting is used to represent cases where the negative mean difference was greater than 0.02 in magnitude.

| Tooth Group | Mean FRI Cat. | Summary Statistics | Adjacent Ages | | | | | |
| --- | --- | --- | --- | --- | --- | --- | --- | --- |
|  |  |  | 9 – 13 | | 13 – 17 | | 17 – 23 | |
| Any Bleaching? | | | No | Yes | No | Yes | No | Yes |
| Early-Erupting | 0.01 – 0.50 | N | 565 | 267 | 345 | 236 | 198 | 147 |
|  |  | Mean  (SD) | -0.17 (0.32) | -0.21 (0.31) | -0.16 (0.31) | -0.17 (0.33) | -0.16 (0.34) | -0.13 (0.33) |
|  | 0.51 – 1.00 | N | 365 | 224 | 187 | 115 | 71 | 58 |
|  |  | Mean  (SD) | -0.50 (0.46) | -0.47 (0.46) | -0.43 (0.55) | -0.43 (0.59) | -0.49 (0.52) | -0.49 (0.47) |
| Late-Erupting | 0.01 – 0.50 | N | - | - | 761 | 434 | 462 | 306 |
|  |  | Mean  (SD) | - | - | -0.18 (0.30) | -0.16 (0.37) | -0.18 (0.30) | -0.16 (0.26) |
|  | 0.51 – 1.00 | N | - | - | 372 | 187 | 91 | 50 |
|  |  | Mean  (SD) | - | - | -0.42 (0.51) | -0.63 (0.32) | -0.60 (0.34) | -0.55 (0.32) |
| Maxillary Incisors | 0.01 – 0.50 | N | 272 | 134 | 216 | 144 | 116 | 87 |
|  |  | Mean  (SD) | -0.13 (0.34) | -0.17 (0.34) | -0.11 (0.34) | -0.11 (0.36) | -0.14 (0.36) | -0.08 (0.31) |
|  | 0.51 – 1.00 | N | 191 | 131 | 97 | 63 | 53 | 42 |
|  |  | Mean  (SD) | -0.46 (0.42) | -0.42 (0.44) | -0.42 (0.48) | -0.46 (0.44) | -0.41 (0.51) | -0.41 (0.48) |

Appendix Table 4 – p-values for the clustered Wilcoxon rank-sum statistic testing for a difference in the distribution of baseline to follow-up mean tooth-level FRI scores between people with and without reported use of any bleaching products. Please note that these p-values were calculated using a version of the Wilcoxon rank-sum test which adjusts for the clustering of teeth within individual participants. These p-values were calculated by approximating the exact test described in Rosner et al. (2006) using 10,000 bootstrap iterations.

| Tooth Group | | Early-Erupting | | Late-Erupting | | Maxillary Incisors | |
| --- | --- | --- | --- | --- | --- | --- | --- |
| Baseline Mean FRI Category | | 0.01 – 0.50 | 0.51 – 1.00 | 0.01 – 0.50 | 0.51 – 1.00 | 0.01 – 0.50 | 0.51 – 1.00 |
| Adjacent Ages | 9 – 13 | 0.45 | 0.71 | - | - | 0.28 | 0.57 |
|  | 13 – 17 | 0.74 | 0.67 | 0.41 | 0.01 | 0.71 | 0.46 |
|  | 17 – 23 | 0.29 | 0.40 | 0.52 | 0.26 | 0.19 | 0.93 |
